# Supplementary material for: The landscape of regulatory genes in brain-wide neuronal phenotypes of a vertebrate brain
Source: eLife. 2021 Dec 13;10:e68224. doi: 10.7554/eLife.68224 (PMC8769648; doi:10.7554/eLife.68224)
Supplement: Transparent reporting form [file elife-68224-transrepform1.docx]

***eLife’s* transparent reporting form**

We encourage authors to provide detailed information *within their submission* to facilitate the interpretation and replication of experiments. Authors can upload supporting documentation to indicate the use of appropriate reporting guidelines for health-related research (see [EQUATOR Network](http://www.equator-network.org/%20)), life science research (see the [BioSharing Information Resource](https://biosharing.org/)), or the [ARRIVE guidelines](http://www.plosbiology.org/article/info:doi/10.1371/journal.pbio.1000412) for reporting work involving animal research. Where applicable, authors should refer to any relevant reporting standards documents in this form.

If you have any questions, please consult our Journal Policies and/or contact us: [editorial@elifesciences.org](mailto:editorial@elifesciences.org).

**Sample-size estimation**

- You should state whether an appropriate sample size was computed when the study was being designed
- You should state the statistical method of sample size computation and any required assumptions
- If no explicit power analysis was used, you should describe how you decided what sample (replicate) size (number) to use

Please outline where this information can be found within the submission (e.g., sections or figure legends), or explain why this information doesn’t apply to your submission:

We collected different cells used individual fish to perform single cell RNA sequencing as Figure 1, Figure 2, and Figure 4;

For single cell morphology analysis at Figure 4, we stated sample size at text, methods, and figure legends.

**Replicates**

- You should report how often each experiment was performed
- You should include a definition of biological versus technical replication
- The data obtained should be provided and sufficient information should be provided to indicate the number of independent biological and/or technical replicates
- If you encountered any outliers, you should describe how these were handled
- Criteria for exclusion/inclusion of data should be clearly stated
- High-throughput sequence data should be uploaded before submission, with a private link for reviewers provided (these are available from both GEO and ArrayExpress)

Please outline where this information can be found within the submission (e.g., sections or figure legends), or explain why this information doesn’t apply to your submission:

For single-cell RNA sequencing samples, we have more than one replicates, as we showed in Figure 1, 2 and 4, in text, methods, and legends. And each replicates were finished at different days.

For morphology analysis, we collect more than 570 individual samples. Each morphological subclass was identified by many samples. The information mentioned in Figure 4.

We identify the gene expression of cells with a criteria of 5% cells or have averaged UMI of each TF-expressed cell UMI = 2 as ON. The information can be found in both sections and figure legends of Figure 1-source data3, Figure 2-source data 3, and Figure 4-source data 1.

High-throughput sequence data have be uploaded to https://bigd.big.ac.cn/gsub/submit/gsa/subCRA002669

**Statistical reporting**

- Statistical analysis methods should be described and justified
- Raw data should be presented in figures whenever informative to do so (typically when N per group is less than 10)
- For each experiment, you should identify the statistical tests used, exact values of N, definitions of center, methods of multiple test correction, and dispersion and precision measures (e.g., mean, median, SD, SEM, confidence intervals; and, for the major substantive results, a measure of effect size (e.g., Pearson's r, Cohen's d)
- Report exact p-values wherever possible alongside the summary statistics and 95% confidence intervals. These should be reported for all key questions and not only when the p-value is less than 0.05.

Please outline where this information can be found within the submission (e.g., sections or figure legends), or explain why this information doesn’t apply to your submission:

We described all statistical analysis methods we used in section and methods of each figures;

We identify the statistical test and exact value of N for Figure 4;

We report the exact p-values for each result, especially in GO analysis at Figure 1-figure supplement 2G, Figure 5A, and Figure 5-figure supplement 1A.

For the statistical analysis of population-level similar pair cluster identification, we use 10% lower as similar, 80% higher as dissimilar, there were shown in Figure 3-figure supplement 2D-E, 4B.

For the differential expression RBPs in each sister pair clusters, we use mean and SEM to describe the results in Figure 5-figure supplement 1B-D.

(For large datasets, or papers with a very large number of statistical tests, you may upload a single table file with tests, Ns, etc., with reference to sections in the manuscript.)

**Group allocation**

- Indicate how samples were allocated into experimental groups (in the case of clinical studies, please specify allocation to treatment method); if randomization was used, please also state if restricted randomization was applied
- Indicate if masking was used during group allocation, data collection and/or data analysis

Please outline where this information can be found within the submission (e.g., sections or figure legends), or explain why this information doesn’t apply to your submission:

In this study, samples were allocated into different groups as we dissected and dissociated specific brain regions in Figure 1; Meanwhile, whole-brain smaples were separated with brain region specific samples;

Different neurotransmitter/neuromodulator type neurons were separate in Figure 2, and Figure 4.

**Additional data files (“source data”)**

- We encourage you to upload relevant additional data files, such as numerical data that are represented as a graph in a figure, or as a summary table
- Where provided, these should be in the most useful format, and they can be uploaded as “Source data” files linked to a main figure or table
- Include model definition files including the full list of parameters used
- Include code used for data analysis (e.g., R, MatLab)
- Avoid stating that data files are “available upon request”

Please indicate the figures or tables for which source data files have been provided:

We reported the regional and neurotransmitter type of each clusters by their origin gene expression as showed in Figure 1- Source Data 3, and Figure 2- Source Data 1.

We identified six major cell types of the whole brain according to gene expression as Figure 1- Source Data 5;

Genes as specific markers for transcriptome-based clusters or morphological classes were showed in Figure 4- Source Data 1 and Figure 4- Source Data 2.

Differentially expressed genes were identified in sister clusters of terminus pairs with divergent pairs showed in Figure 5- Source Data 1.
